# An alternative dosing strategy for ropeginterferon alfa-2b may help improve outcomes in myeloproliferative neoplasms: An overview of previous and ongoing studies with perspectives on the future

**DOI:** 10.3389/fonc.2023.1109866

**Published:** 2023-01-19

**Authors:** Albert Qin, Raymond W. Urbanski, Lennex Yu, Tasfia Ahmed, John Mascarenhas

**Affiliations:** ^1^ PharmaEssentia Corporation, Taipei, Taiwan; ^2^ PharmaEssentia USA Corporation, Burlington, MA, United States; ^3^ Tisch Cancer Institute, Division of Hematology and Medical Oncology, Icahn School of Medicine at Mount Sinai, New York, NY, United States

**Keywords:** ropeginterferon alfa-2b, myeloproliferative neoplasm, polycythemia vera, alternative dosing strategy, clinical study, interferon, pegylated interferon

## Abstract

Ropeginterferon alfa-2b is a novel, long-acting mono-pegylated proline-IFN-alpha-2b approved for treatment of polycythemia vera in adults, regardless of thrombotic risk level or treatment history. Clinical trial data indicate the dose and titration of ropeginterferon alfa-2b is safe and effective. However, additional studies may provide rationale for an amended, higher initial dosage and rapid titration. This article is an overview of current and upcoming studies of ropeginterferon alfa-2b in myeloproliferative neoplasms that support the exploration of an amended dosing scheme in order to optimize patient tolerability and efficacy outcomes.

## Introduction

Polycythemia vera (PV), essential thrombocythemia (ET), and myelofibrosis (MF) including pre-fibrotic/early primary myelofibrosis (PMF) are classical Philadelphia chromosome-negative myeloproliferative neoplasms (MPNs) characterized by the uncontrolled clonal proliferation of hematopoietic stem or progenitor cells due to driver mutations of genes including *JAK2*, *CALR*, or *MPL* (1, 2). Thrombohemorrhagic complications and leukemic transformations are part of the natural history of MPNs (3, 4). Most PV patients harbor a *JAK2* mutation, the vast majority being a point mutation on exon 14 (V617F) (4, 5). PV is considered an inflammatory neoplasm (4, 6) characterized by clonal erythrocytosis, often accompanied by thrombocytosis and leukocytosis, leading to a high risk of thromboembolic events (7).

Interferon (IFN) alfa-based therapies demonstrate preferential activity against neoplastic hematopoietic stem or progenitor cells and elicit complete and durable hematologic remission and molecular response, reduce PV progressive events, and shows improvement of patient myelofibrosis-free and overall survival (8–13). Ropeginterferon alfa-2b is a novel mono-pegylated IFN with pharmacokinetic properties allowing dosing once every 2 to 4 weeks (14–18). It was approved by the US Food and Drug Administration (FDA) in November 2021 and the European Medicines Agency (EMA) in February 2019 for adults with PV and is the first and only IFN approved for PV treatment (19, 20). The NCCN Guidelines place ropeginterferon alfa-2b as a treatment option for low (Category 2B) and high-risk PV patients (Category 2A) (2). The European LeukemiaNet (ELN) recommends ropeginterferon alfa-2b and pegylated IFN alfa-2a as a therapeutic option for treatment naive patients with low-risk PV requiring cytoreductive therapy (21).

The safety and efficacy of ropeginterferon alfa-2b was assessed in the pivotal phase 1/2 PEGINVERA, phase 3 PROUD-PV, and its extension CONTINUATION-PV studies (11, 12, 15, 22). Doses in these studies ranged from 50 micrograms (mcg) to 540 mcg every two weeks, leading to the recommended starting dose of 100 mcg [50 mcg for patients receiving hydroxyurea (HU)], and increasing 50 mcg every two weeks to a maximum of 500 mcg, until hematological parameters are stabilized (hematocrit <45%, platelets <400 x 10^9^/L, WBC <10 x 10^9^/L) (14). The dosing interval may increase to 4 weeks upon achieving hematological stability for at least one year on a stable dose (14). Since these studies were reported, additional clinical investigation into alternative dosing strategies suggest a potential role for rapid titration and higher starting doses of ropeginterferon alfa-2b.

The safety of ropeginterferon alfa-2b was also evaluated in patients with chronic hepatitis B or hepatitis C (genotypes 1 and 2) at doses ranging from 270 to 450 mcg every 2 weeks as monotherapy or in combination with ribavirin (23–27), or in COVID-19 patients in combination with standard of care at 250 mcg (28). Most reported adverse events (AEs) during ropeginterferon alfa-2b treatment were mild or moderate and toxicities ≥grade 3 were uncommon. These results suggest tolerability and safety of higher starting doses of ropeginterferon alfa-2b.

In this article, we review the clinical development of ropeginterferon alfa-2b in PV and current research extending into other related MPNs in order to closely examine a critical connection between an amended dosing schema and key disease outcome measures such as a reduction in thromboembolic risk, complete hematologic response, and reduction in the driver mutation variant allele frequency (VAF), while maintaining adequate safety and tolerability.

## Polycythemia vera

### Clinical trial experience

The PEGINVERA study was a phase 1/2 multicenter study conducted to evaluate the dosing, tolerability, and efficacy of ropeginterferon alfa-2b in PV patients (15). A diverse PV patient population was enrolled including newly diagnosed, those pretreated with HU, and those receiving HU, who were at low- or high-risk for thromboembolic events. Eight subcutaneously administered dose levels (50, 100, 150, 225, 300, 360, 450, and 540 mcg) given every 2 weeks were explored. The Phase 1 portion of the study aimed to identify the maximum tolerated dose (MTD) of ropeginterferon alfa-2b in 25 patients using a 3 + 3 dose-escalation method. No dose-limiting toxicities (DLTs) were observed and the MTD was determined to be the highest dose level that was evaluated, or 540 mcg. The Phase 2 portion of the PEGINVERA study was designed to assess efficacy in the form of complete response (CR) including hematologic remission according to modified ELN criteria (**Table 1**) and molecular response (MR) in 26 patients with dose titrations based on disease response and tolerability. After a median time of 5 years of treatment, results from this study showed that 27 of 42 patients (64.3%) attained a complete hematologic response (CHR) from the efficacy analysis set. It required a median time of 34 weeks of treatment for patients to achieve a CHR (22). For *JAK2*V617F VAF, 12/42 patients (28.6%) achieved a complete molecular response (CMR) as the best observed response. A median time of 82 weeks was required for patients to achieve a CMR and 34 weeks to achieve any MR. The study demonstrated that ropeginterferon alfa-2b treatment for up to 7 years was efficacious and well-tolerated (22).

**Table 1:** Measured response definitions for the clinical studies of ropeginterferon alfa-2b for the PV treatment.

	Complete response (CR)	Partial response (PR)	Complete Molecular Response (CMR)	Partial Molecular Response
PEGINVERA	hematocrit < 45% without phlebotomy in the past 2 months, platelet count ≤ 400 x 10^9^/L, white blood cell count (WBC) ≤ 10 x 10^9^/L, normal spleen size on ultrasound imaging, and the absence of thromboembolic events^a^	Defined as either hematocrit < 45% without phlebotomy but with persistent splenomegaly or platelets >400x10^9^/Lor reduction of phlebotomy requirements by at least 50%	Reduction of JAK2 allelic burden from DNA samples to undetectable levels.	Defined as 1) a reduction of ≥50% from baseline value in patients with <50% mutant allele burden at baseline, OR 2) reduction of ≥25% in patients with >50% mutant allele burden at baseline.
PROUD-PV and CONTINUATION-PV	1) CHR^b^ with normal spleen size 2) CHR with improved disease burden (i.e., splenomegaly, microvascular disturbances, pruritus, and headache) 3) CHR^b^	Not Reported	Reduction of any molecular abnormality to undetectable levels	Reduction in the JAK2 V617F allele burden of at least 50% from baseline (if the baseline value is <50%) and a reduction of at least 25% from baseline (if the baseline level is at least 50%)
Phase 2 Japan	CHR: hematocrit < 45%, WBC count ≤ 10 x10^9^/L, and platelet count ≤ 400 x10^9^/L; no phlebotomy in the previous 12 weeks			
Ongoing Studies				
Phase 2 China	CHR: hematocrit <45% without phlebotomy or erythrocyte apheresis in the preceding 12 weeks, platelet ≤400×10^9^/L, leukocyte ≤10×10^9^/L			
IIT Korea	CHR (hematocrit <45% without phlebotomy in the previous 12 weeks, platelet ≤400×10^9^/L, leukocyte ≤10×10^9^/L) and MR			

CHR, complete hematologic response, IIT, investigator-initiated trial.

a CR for PEGINVERA was defined according to the modified ELN (European Leukemia Net) criteria.

b CHR defined as hematocrit < 45% without phlebotomy in the past 3 months, platelet count ≤ 400 x 10^9^/L, WBC ≤ 10 x 10^9^/L.

The phase 3 PROUD-PV study (N=254) enrolled early-stage patients diagnosed with PV who were either HU naïve or those who had been treated with HU for <3 years (11, 12). Patients underwent stratified randomization by age, history of thrombosis, and prior use of HU, and then randomized to ropeginterferon alfa-2b or HU group. The primary endpoint was disease response rate after 12 months of therapy. Dose initiation and titration were similar to the PEGINVERA Phase 2 trial. Ropeginterferon alfa-2b treatment was started at a dose of 100 mcg or 50 mcg for patients receiving HU and the dose increased by 50 mcg every 2 weeks for the maximum dose of 500 mcg. It was estimated that the mean efficacious dose was reached after approximately 16.2 weeks (95% CI 14·8–17·6) for ropeginterferon alfa-2b and 11.4 weeks (95% CI 10·2–12·6) for HU (11). At 12 months, the PROUD-PV study failed to demonstrate superiority regarding the primary endpoint (ropeginterferon alfa-2b 21% vs. HU 28%) and secondary endpoints of CHR (ropeginterferon alfa-2b 43% vs. HU 46%) and MR (ropeginterferon alfa-2b 34% vs. HU 42%) (11). It is unknown if the difference in time to reach an effective dose or an optimal maximum dose plateau contributed to the observed outcomes.

The extension study, CONTINUATION-PV, recruited 171 patients who completed the PROUD-PV study (95 in the ropeginterferon alfa-2b group vs. 76 in control) (11). While the control group was allowed to change treatment from HU to best available treatment (BAT), 64/66 patients (97%) remained on HU treatment. The dose for ropeginterferon alfa-2b used in PROUD-PV was continued at the discretion of the investigator and administered every 2, 3, or 4 weeks. Ropeginterferon alfa-2b group showed a continuous trend of improved outcomes. At 36 months, ropeginterferon alfa-2b treatment led to CHR in 67 of 95 patients (71%) compared to 38 of 74 (51%) in the HU/BAT group (p=0·012). The ropeginterferon alfa-2b group showed a superior rate in CHR with improved disease burden than the HU/BAT group (53% versus 38%; p=0·044) (11). A consistent reduction of median *JAK2*V617F allele burden was observed at every timepoint starting from screening (38%) to month 60 (8%) (12).

A multicenter, 52-week single-arm study evaluated the safety and efficacy of ropeginterferon alfa-2b in 29 Japanese patients diagnosed with PV (29). This trial followed the same titration regimen as the PROUD-PV study. The primary outcome was a durable CHR at 9 and 12 months and it was achieved in 8/29 patients (28%). At 12 months, 51% of patients achieved CHR, similar to 43% at 12 months reported in PROUD-PV. One serious treatment emergent AE (TEAE) of gastroenteritis was reported but was not deemed treatment-related. All patients experienced at least one TEAE and none were grade ≥3 in severity. An extension of the study continues (NCT04655092) (30) and its results may confirm the long-term effect of ropeginterferon alfa-2b observed in the PROUD/CONTINUATION-PV study.

A phase 2b, multicenter, open-label, parallel-group, randomized study evaluated the safety and efficacy of ropeginterferon alfa-2b at a fixed dose of 100 mcg every 2 weeks with phlebotomy and aspirin compared to phlebotomy and aspirin alone in 127 patients with low-risk PV (31). The primary endpoint was the maintenance of median hematocrit <45% during a 12-month period without disease progression. Following treatment, 84% of patients in the experimental group achieved the primary end point versus 60% in the control group (CI: 7-41%; p=0.0075). No disease progression was noted in the ropeginterferon alfa-2b group compared to 8% in the control. There was no statistically significant difference between the groups in frequency of AEs of grade 3/4 observed.

### Ongoing investigational trial experience

Based on the hematological outcomes observed in the clinical trials for ropeginterferon alfa-2b, several international investigational studies were designed to further understand the relationship between short and long-term outcome measures and safety with an amended, and potentially optimized dosing regimen in PV patients.

An ongoing, phase 2, single-arm study in 49 Chinese PV patients who are resistant or intolerant to HU is evaluating the safety and efficacy of ropeginterferon alfa-2b when administered in a 250-350-500 mcg dosing titration regimen (32, 33). In this dosing scheme, the starting dose is 250 mcg, followed by 350 mcg at Week 2, and a target dose of 500 mcg at Week 4. Interim results reveal a 52% CHR rate at Week 24, comparable to 43% at Week 52 observed in PROUD-PV. *JAK2*V617F VAF decreased over time with two patients achieving a level <3%. TEAEs were reported in >95% of patients with possible treatment-related Grade ≥ 3 AEs in five patients (10.2%). This suggests that the more aggressive dosing schema results in a more rapid time to CHR.

A single-arm open-label, multicenter study in South Korea is currently evaluating the safety, efficacy, and tolerability of 250-350-500 mcg ropeginterferon alfa-2b dosing in PV patients who are either HU naïve or previously treated (34). Only 4.4% of patients at an interim data-cut required dose reductions during dose escalation, indicating good tolerability of this dosing approach. No treatment-related serious AEs were reported, and the majority of the AEs were grade 1 or 2. At the interim data-cut, 45, 20, and 6 patients were evaluable at 3, 6, and 9 months, respectively. The mean hematocrit, platelet, and WBC counts steadily decreased from baseline to Month 6 and 9. The *JAK2*V617F VAF showed a trend of rapid decrease with treatment from 59.7% at baseline to 42.4% at Month 3, and 37.1% at Month 6. The data suggests that ropeginterferon alfa-2b therapy with the 250-350-500 mcg dosing regimen induced hematological and molecular responses and was well-tolerated in Korean patients with PV (34).

ECLIPSE-PV (NCT05481151) is a phase 3b study to assess the efficacy, safety, and tolerability of ropeginterferon alfa-2b in North American adult PV patients utilizing the 250-350-500 mcg regimen (35, 36). It is hypothesized that the response rates of PROUD/CONTINUATION-PV will be observed in a shorter period of time (36).

## Clinical experience of ropeginterferon alfa-2b in other MPNs

Decades of clinical research conducted with IFN alfa support its role as a treatment option across MPNs (37–40). While ropeginterferon alfa-2b is not currently approved to treat ET or MF, the existing IFN alfa data support further clinical evaluation of ropeginterferon alfa-2b for other MPN diagnoses (**Table 2**).

**Table 2:** Overview of clinical studies with ropeginterferon alfa-2b.

First author [Reference]	Publication type	Year	Study name	Discipline	Disease	Phase	Dosing regimen	Maximum dose (mcg)
Gisslinger (15)	Original article	2015	PEGINVERA	Hematology/Oncology	Polycythemia vera (PV)	I/II	Starting dose at 100 mcg or 50 mcg (under HU treatment). Increase 50 mcg every 2 weeks	540
Gisslinger (11)	Original article	2020	PROUD/CONTI-PV	Hematology/Oncology	PV	III	As above	500
Kiladjian (12)	Letter to editor	2022	PROUD/CONTI-PV	Hematology/Oncology	PV	III	As above	500
Edahiro (29)	Original article	2022	NCT04182100	Hematology/Oncology	PV	II	As above	500
Jin (32)	Conference abstract	2022	NCT05485948	Hematology/Oncology	PV	II	250 mcg as the starting dose, 350mcg at Week 2, then 500 mcg at week 4	500
Lee (34)	Conference abstract	2022	N/A	Hematology/Oncology	PV	II	As above	500
Barbui (31)	Original article	2021	Low-PV	Hematology/Oncology	PV	II	Fixed dose at 100 mcg	100
Verstovsek (41)	Review	2022	Surpass-ET	Hematology/Oncology	ET	III	250 mcg as the starting dose, 350mcg at Week 2, then 500 mcg at week 4	500
Gisslinger (42)	Conference abstract	2018	N/A	Hematology/Oncology	Myelofibrosis (MF) (prefibrotic/early)	II	50 to 200 mcg every 4 weeks	200
Palmer (43)	Conference abstract	2018	NCT02370329	Hematology/Oncology	MF	II	50 mcg with dose titration to 300 mcg every 2 weeks	300
Gill (44)	Conference abstract	2022	NCT04988815	Hematology/Oncology	MF (prefibrotic/early)	II	250 mcg as the starting dose, 350mcg at Week 2, then 500 mcg at week 4	500
Chen (45)	Conference abstract	2019	CUP	Hematology/Oncology	PV, ET, and MF	N/A	250 mcg as the starting dose, 350mcg at Week 2, then 500 mcg at week 4	500
Huang (46)	Original article	2020	CUP	Hematology/Oncology	PV, ET, and MF	N/A	250 mcg as the starting dose, 350mcg at Week 2, then 500 mcg at week 4	500
Huang (23)	Original article	2020	N/A	Hepatology	Hepatitis B	I/II	Fixed dose at 350 or 450 mcg	450
Hsu (24)	Original article	2021	N/A	Hepatology	Hepatitis C	II	Fixed dose at 270, 360 or 450 mcg	450
Lin (25)	Original article	2021	NCT01587586	Hepatology	Hepatitis C	II	Fixed dose at 180, 270 or 450 mcg	450
Chen (28)	Original article	2022	CUP	Infectious disease	Coronavirus disease 2019	N/A	Single dose at 250 mcg	250
Huang (18)	Original article	2021	N/A	Pharmacokinetics	Healthy participants	I	Single dose at 90, 180, and 270 mcg	270
Huang (17)	Original article	2022	NCT05129644	Pharmacokinetics	Healthy participants	I	Single doses ranging from 24 to 270 mcg	270
Miyachi (16)	Original article	2021	NCT03546465	Pharmacokinetics	Healthy participants	I	Single dose at 100, 200, or 300 mcg	300

CUP, compassionate use program; N/A, not applicable; NR, not reported.

The ongoing global phase 3, multicenter, randomized, controlled SURPASS-ET trial (NCT04285086) (41), and the single-arm EXCEED-ET trial (NCT05482971) in US and Canada (47), are currently evaluating the 250-350-500 mcg dosing scheme. Previous exploratory clinical research and meta-analysis already indicated that interferon therapy could be a safe and effective treatment for ET (42). This Phase 3 trial aims to provide pivotal data to support the approval of ropeginterferon alfa-2b for the treatment of ET.

A phase 2 study evaluated the efficacy of ropeginterferon alfa-2b in 25 patients with pre-fibrotic PMF (43). Ropeginterferon alfa-2b was administered at doses between 50 to 200 mcg every 4 weeks during the 24-month treatment period in the treatment-naïve group. Clinical improvements were observed, and no patient showed disease progression at 2 years. Two patients withdrew from the study one year after starting ropeginterferon alfa-2b treatment due to psychological related AEs (43). A second study recruited 8 MF patients (2 early and 6 intermediate/high-risk) who received 50 mcg with dose titration to 300 mcg every two weeks (44). Preliminary clinical improvement regarding spleen size and symptom scores was observed. One patient discontinued therapy due to dizziness and atrial fibrillation (44).

An ongoing, phase 2 investigator-initiated trial assessing the efficacy and safety of the 250-350-500 mcg dosing titration of ropeginterferon alfa-2b in early MF (NCT04988815), includes 56 patients with pre-fibrotic/early MF, overt PMF, post-PV MF or post-ET MF, and low/intermediate - 1 risk category according to dynamic international prognostic scoring system (DIPSS) (46). Interim results indicate 71% of treated patients at Week 12 and 67% at Week 24 achieved clinico-hematologic complete response. Of 39 patients harboring *JAK2* V617F, 36 reported reductions in VAF at 24 weeks with a reduction >50% in three treated patients and undetectable levels in one patient. No progression to overt MF or blast phase disease was observed. No treatment discontinuation, new safety signals, or deaths were reported thus far. The treatment options for patients with early MF including pre-fibrotic PMF have been very limited. The data from this study suggest that ropeginterferon alfa-2b potentially provides an effective treatment option for patients with early MF.

Further, a compassionate use program (CUP) recruited HU and/or anagrelide-resistant or intolerant MPN patients and administered ropeginterferon alfa-2b mostly using the 250-350-500 mcg dosing regimen in Taiwan (45). Published data from an initial cohort of nine patients show tolerability and substantial efficacy. Additional data in 20 MPN patients (14 PV, 4 ET, 1 post-ET MF, 1 pre-fibrotic PMF) further demonstrate tolerability and efficacy (45). At Week 52, eight of 11 response-evaluable PV patients (72.7%) achieved CHR and two of three ET patients (66.7%) achieved CHR. The median time to CHR was 27 and 24 weeks for PV and ET, respectively.

Overall, ropeginterferon alfa-2b treatment of MPNs at the 250-350-500 mcg dosing regimen appears to lead to notable and rapidly occurring clinical efficacy or activities with tolerability. The drug-related adverse events were generally well-tolerated and manageable, indicating a favorable benefit-risk profile.

## Discussion

The response outcomes observed in multiple clinical trials of ropeginterferon alfa-2b confirm clinical benefits in MPN patients. Emerging data utilizing the 250-350-500 mcg regimen suggest that a higher initial dose with faster dose titration may lead to earlier complete hematological remission. Although a direct comparison is not possible in the absence of head-to-head data, treatment with this regimen in PV for 6 months led to CHR rates comparable to those observed at 12 months in the studies utilizing the low starting dose and slower titration schema.

Support for a higher starting dose of ropeginterferon alfa-2b comes from the outcomes noted in the PROUD-PV and the study by Edahiro et al. compared to the outcomes from the emerging data of the 250-350-500 mcg ropeginterferon alfa-2b titration regimen from several recent sources including a CUP in Taiwan (46, 48), a Phase 2 study in China (32, 33), an IIT study in Korea (34). For example, the study by Edahiro et al. followed the slow titration regimen, whereas the 14 patients with HU and/or anagrelide resistance or intolerance enrolled in the CUP study followed the 250-350-500 mcg titration regimen, and the median time to response was 52 weeks vs 27 weeks. At 52 weeks, patients from Edahiro et al. study had a CHR rate of 51% compared to 73% in the CUP study. Interim results from the Chinese Phase II study in HU-resistant or intolerant PV patients showed a CHR rate of 52% at 24 weeks (32). The CHR rate at 24 weeks (6 months) was even notably higher than the rate of 43% observed at 12 months in the PROUD-PV study. The data from the Korean IIT study in HU naïve or pre-treated PV patients also indicate higher hematologic and molecular responses at 6 months (34).

Indeed, the risk of a thromboembolic event is highest immediately before or after establishing the diagnosis of PV (3, 49–51). A potential risk of thrombosis may not be adequately addressed by the low starting dose and slow titration regimen as time to hematologic response is delayed. Furthermore, it is widely known that high *JAK2*V617F VAF poses greater risk of myelofibrotic transformation (52–54), and that high *JAK2*V617F VAF is associated with high leukocyte count (55, 56) which has also been implicated as an independent risk factor for thromboembolic events (57, 58). Results from ongoing clinical studies utilizing the 250-250-500 dosing regimen will provide key insights into the correlation between dosage and outcome response and rates of thromboembolic complications. Thromboembolic complications have not been reported during the intra-patient dose escalations with the 250-250-500 dosing regimen from the existing data. Given that it has only three step dose escalations with a higher starting dose, it is reasonable to believe that the dosing regimen might potentially minimize the risk of thrombosis and hemorrhage associated with an under-dosing during dose titrations. Although a thromboembolic risk associated with an under-dosing during the slow dose titrations in PV patients could potentially be managed with phlebotomies, the 250-350-500 dosing regimen of ropeginterferon alfa-2b provides a treatment option of leveraging the risk with rapid induction of hematologic remission and molecular response associated with VAF decreases of drive mutations such as *JAK2*V617F in broader MPN patients. Therefore, the importance of an optimal dosing and titration schedule for ropeginterferon alfa-2b may need to be further explored to better understand its potential impact on critical efficacy and tolerability outcomes in PV or other MPNs.

## Data availability statement

The original contributions presented in the study are included in the article/supplementary material. Further inquiries can be directed to the corresponding author.

## Author contributions

All authors listed have made a substantial, direct, and intellectual contribution to the work and approved it for publication.
